# Editorial: The Bone Marrow Niche in Normal and Malignant Haematopoiesis

**DOI:** 10.3389/fcell.2022.870114

**Published:** 2022-02-28

**Authors:** Michela Colombo, Ruggiero Norfo, Giada Bianchi, Aldo M. Roccaro

**Affiliations:** ^1^ Haematopoietic Stem Cell Biology Laboratory, Medical Research Council (MRC) Weatherall Institute of Molecular Medicine, University of Oxford, Oxford, United Kingdom; ^2^ MRC Molecular Haematology Unit, MRC Weatherall Institute of Molecular Medicine, Radcliffe Department of Medicine, Medical Sciences Division, University of Oxford, Oxford, United Kingdom; ^3^ Dana–Farber Cancer Institute, Boston, MA, United States; ^4^ Clinical Research Development and Phase I Unit, ASST Spedali Civili di Brescia, Brescia, Italy

**Keywords:** bone marrow, microenviroment, haematology, cancer, cancer biology

The bone marrow (BM) microenvironment is composed by distinct compartments with a specific cellular composition and tissue organization, where the different haematopoietic stem and progenitor cells reside, are maintained, and differentiate into multiple blood lineages (Yu and Scadden, 2016; Comazzetto et al., 2021).

Several studies have demonstrated that these niches have a key role in the regulation of different steps of haematopoiesis. Indeed, the crosstalk with nearby cells (such as mesenchymal stem cells, fibroblasts, endothelial cells, adipocytes, immune and bone cells) influences the behaviour of haematopoietic stem cells (HSCs) and contributes to their self-renewal, proliferation, lineage commitment, differentiation, and mobilization (Morrison and Scadden, 2014; Sarkaria et al., 2018).

Given the crucial role of the BM stromal cells in regulating haematopoiesis under physiological condition, it is not surprising that alterations within the BM niche have been shown to play an active role in the development and progression of haematological malignancies (Méndez-Ferrer et al., 2020).

Within this Research Topic, we have gathered several contributions that analyse the interactions between haematopoietic cells and the BM niche, with a main focus on how they are subverted by haematologic tumours, in order to promote the survival and expansion of malignant cells.

Understanding the molecular mediators of normal haematopoiesis is crucial for the improvement of regenerative medicine and cancer therapies. Here, Ferrera Pissarra et al. explore the role of ARHGAP21 (a member of the RhoGAP family involved in cell adhesion, growth and differentiation) in the marrow microenvironment. Comparing the BM of Arhgap21^+/−^ and wild type mice, they detected an increase in the frequency of osteoblastic lineage cells, while there were no differences in the frequencies of endothelial cells and multipotent stromal cells. Consistently, the BM of Arhgap21^+/−^ mice is characterized by higher levels of osteocalcin, and BM cells showed increased expression of Col1a1 and Ocn and decreased expression of Trap1 after osteogenic differentiation with increased capacity of generating CFU-OB (osteogenic colony-forming units) *in vitro*.

On the other hand, Arhgap21^+/−^ mice transplanted with normal BM cells showed leukopenia during transplantation recovery, suggesting that ARHGAP21 could have a role in balancing the composition of the BM microenvironment and in determining its ability to support haematopoiesis.

Recently, the development of single cell technologies has allowed to systematically identify distinct bone marrow cell types, giving us the first insights on the molecular and spatial composition of the BM niche, the relationships between the different cell types, and how they are altered by tumour development (Al-Sabah et al., 2020).

Herein, Woods et al. provide us with an overview of the composition of the different niches within the BM and summarised recent single-cell studies that characterized both the murine and the human the BM-mesenchymal stromal cells (MSCs) biology, identifying several hierarchical subsets with specific transcriptional profiles and functions. They then focused specifically on osteo-adipogenic progenitors and on their role in the regulation of haematopoiesis both in homeostasis, aging or during the development of myeloid malignancies. Finally, due to the crucial role of MSCs in HSC maintenance and in leukaemia cell growth, they also examined the therapeutic potential of MSCs for rejuvenation of the niche and their potential anti-tumour ability.

MSCs contribution to the homeostasis of the BM depends also on their metabolism (Hawkes and Mostoufi-Moab, 2019). Chiu et al. summarize the role of certain amino acids in the complex interactions existing between MSC and the surrounding cells, with a particular emphasis on immune cells and malignant haematopoietic cells. Specifically, they focused on the possibility to target this crosstalk by interfering with amino acid metabolism or intercellular fluxes in different haematopoietic cancers, such as T-ALL, B-ALL, CLL and AML.

Acute Myeloid Leukaemia (AML) remains a high-risk haematologic malignancy, with a 5 years overall survival rate of around 20% despite the development of novel therapies (Pimenta et al., 2021). Acquisition of drug resistance is a major clinical hurdle in the care of AML patients. Miari et al. discussed the different mechanisms of BM-driven drug resistance in AML, focusing on the pathophysiological role of leukaemia-supporting macrophages. Indeed, single cells technologies have given us the opportunity to more accurately dissect the different subpopulations of macrophages, and to better understand which population should be targeted in order to tackle drug resistance in AML. Macrophages have been shown to promote the activation of anti-cell death pathways in AML cells, and different macrophage phenotypes can be associated with clinical outcomes in AML, conferring them a potential prognostic value.

Given the poor clinical outcome of AML patients, finding therapeutic options is key. Marconi Roversi et al. investigated the role of Haematopoietic Cell kinase (HCK) in mediating the activity of the CXCL12/CXCR4 pathway in AML, with the final goal to provide the rational for the development of anti-HCK therapies for this disease. The authors demonstrated that the inhibition of HCK can hamper CXCL12-driven migration of both leukemic cell lines and CD34 ^+^ cells isolated from AML patients’ bone marrow, through a decrease of the activation of the CXCL12/CXCR4/MAPK/ERK and CXCL12/CXCR4/PI3K/AKT signalling, and by causing a lower rate of actin polymerization, that reduced cytoskeleton dynamics.

AML is not the only haematological disease in which alterations within the BM niche promote tumour cells growth, disease progression and overall patient outcome. In their review, Jalali and Ansell focus on both cellular as well as non-cellular BM compartments and on how they are altered across a variety of aggressive and indolent lymphoid malignancies, including mantle cell lymphoma, diffuse large B-cell lymphoma, Waldenstrom Macroglobulinemia and follicular lymphoma, discussing strategies of intervention and their potential therapeutic impact in clinical settings.

One of the main players in the re-shaping the BM morphology is inflammation, that can change BM cellular composition and promote the development of haematological malignancies (Mitroulis et al., 2020). Fröbel et al. review how inflammatory stimulus, acute stress and ageing are able to alter the composition of the BM, changing both the frequency of the different cell types and the secreted factors that are present in the niche. In their paper, they also described how these alterations may contribute to the development of chronic malignancies (i.e., myelodysplastic syndrome and myeloproliferative neoplasm) and how these conditions are able to re-shape the BM niche. Furthermore, in order to study the cross-talk between niche cells and haematopoietic cells *in vivo*, xenograft models are key. In their review, Fröbel and others discuss which soluble factors, produced from the murine BM niche, can support human HSC engraftment and function in mice.

In conclusion, the present Research Topic has collected several contributions on the multifaceted role of the bone marrow microenvironment in the regulation of haematopoiesis and in the development and progression of blood cancers. The increasing knowledge in the field is paving the way towards novel therapeutic strategies that will be able to uncouple the pathological crosstalk between the different cells compartments and to finally improve the outcomes of these diseases.
